# Impact of tumor size and cirrhotic background for differentiating HCC and ICC with CEUS: does it matter for patients undergoing hepatectomy?

**DOI:** 10.18632/oncotarget.19624

**Published:** 2017-07-27

**Authors:** Chen Jin, Xiao-Yun Zhang, Jia-Wu Li, Chuan Li, Wei Peng, Tian-Fu Wen, Yan Luo, Qiang Lu, Xiao-Fei Zhong, Jing-Yi Zhang, Lv-Nan Yan, Jia-Yin Yang

**Affiliations:** ^1^ Department of Liver Surgery and Liver Transplantation Center, West China Hospital, Sichuan University, Chengdu, Sichuan Province, China; ^2^ Department of Sonography, West China Hospital, Sichuan University, Chengdu, Sichuan Province, China

**Keywords:** contrast-enhanced ultrasound, intrahepatic cholangiocarcinoma, hepatocellular carcinoma, differential diagnosis, clinical application

## Abstract

**Objectives:**

The aim of this study was to investigate the role of contrast-enhanced ultrasound (CEUS) in differentiating hepatocellular carcinoma (HCC) *vs.* intrahepatic cholangiocarcinoma (ICC) and primary liver cancer *vs.* benign liver lesions for surgical decision making.

**Methods:**

Data from 328 patients (296 primary liver cancer patients: 232 HCC and 64 ICC patients and 32 benign hepatic lesion patients) who underwent hepatectomy at our center were retrospectively collected from 2010 to 2015. Conventional ultrasound (US) and CEUS were performed for all patients before hepatectomy. Enhancement patterns in CEUS were classified and compared for HCC *vs*. ICC and for primary liver cancer *vs*. benign lesions.

**Results:**

Primary liver cancer and hepatic benign lesions could be distinguished by CEUS in different phases. The most obvious differences were in the portal and delayed phases, in which benign lesions could still show hyperenhancement (46.9% *vs*. 0.0% and *p* < 0.001 in the portal phase; 43.7% *vs*. 0.0% and *p* < 0.001 in the delayed phase). For differentiating HCC and ICC, our results revealed that HCC and ICC displayed different enhancement patterns in the arterial phase (*p* < 0.001) and the portal phase (*p* < 0.001). In the subgroup analyses, both HCC and ICC showed a high rate of homogeneous hyperenhancement during the arterial phase when tumors were ≤5 cm (87.2% *vs*. 64.0% and *p* = 0.008) or the Ishak score was ≥5 (75.8% vs. 42.9% and *p* = 0.023), although there was statistical difference. However, during the portal phase, ICC > 5 cm showed significantly more frequent hypoenhancement (92.3% *vs*. 54.5% and *p* < 0.001) and less isoenhancement (7.7% *vs*. 45.5% and *p* < 0.001) than HCC; additionally, during the portal phase, there was no statistical difference in the enhancement patterns of ICC with different hepatic backgrounds.

**Conclusions:**

Tumor size and hepatic background should be taken into consideration when distinguishing HCC and ICC before surgery. However, CEUS is a helpful tool for differentiating malignant and benign hepatic lesions. For patients who require surgical treatment, CEUS may help with surgical decision making.

## INTRODUCTION

Contrast-enhanced ultrasound (CEUS) has been utilized to characterize hepatic lesions for over 10 years because its offers a diagnostic accuracy range comparable to that of contrast computed tomography (CT) and contrast magnetic resonance imaging (MRI) [1]. CEUS using the contrast agent Sonovue, which is a blood pool agent, can better reveal the hemodynamic features of hepatic lesions and provide clearer information during the delayed phase [2]. As previous studies have demonstrated [3–6], imaging features reflect pathologic information, which could be a reason why imaging every phase with CT, MRI or CEUS can help to differentially diagnose suspicious hepatic lesions.

In addition, CEUS, together with contrast CT and contrast MRI, was recommended for the non-invasive diagnosis of hepatocellular carcinoma (HCC) in 2005 by the American Association for the Study of Liver Diseases (AASLD) [7]. However, its ability to differentially diagnose HCC and intrahepatic cholangiocarcinoma (ICC) smaller than 3 cm with a cirrhotic background was met with incredulity [8]. Thus, the use of CEUS as a technique for diagnosing hepatic nodules in cirrhosis was dropped from the updated AASLD guidelines in 2011 [9] and from the guidelines of the European Association for the Study of the Liver (EASL) in 2012 [10]. Several studies analyzed the use of CEUS to differentiate ICC from HCC according to tumor size and hepatic background [8, 11–14]; however, few of these studies discussed the size of the nodules and cirrhosis, and the number of patients in those studies was relatively small. In addition, comparisons of primary liver cancer and benign tumors and the application of CEUS for treatment aspects were rarely mentioned.

Therefore, the aim of our study was to investigate the impact of tumor size and cirrhotic background on the ability to differentiate HCC and ICC using CEUS, to analyze the enhancement pattern differences of malignant *vs*. benign tumors, and to examine the clinical application of CEUS for surgical decision making.

## RESULTS

### Patient characteristics

A total of 328 patients were enrolled in our study (Table 1) after the inclusion and exclusion criteria of our study were applied. The patients comprised 296 primary liver cancer patients (232 HCC patients and 64 ICC patients) and 32 benign hepatic lesion patients (20 hemangioma patients, 3 parasitization patients, 5 focal nodular hyperplasia (FNH) patients and 4 liver abscess patients).

**Table 1:** The baseline of patients who were diagnosed primary liver cancer and benign lesions

Variables	Primary liver cancer(n=296)	Hepatic benign lesions(n=32)	*P*
Age(years)	52.84±11.58	45.25±12.12	<0.001
Male/female(n)	223:73	43:21	0.209
Etiology (n):			
Hemangioma	-	20(62.5%)	
Parasitization	-	3(9.4%)	
Focal nodular hyperplasia (FNH)	-	5(15.6%)	
Liver abscess	-	4(12.5%)	
Child-Pugh A	296(100%)	32(100%)	
Hemoglobin(g/l)	141.60±19.51	137.68±17.42	0.278
Platelets (10^9^/L)	145.40±71.96	188.91±67.10	0.001
White blood cell (10^9^/L)	6.30±5.24	5.97±1.89	0.731
Total bilirubin (umol/L)	15.79±11.98	13.97±5.94	0.396
ALT (IU/L)	44.99±41.22	32.91±24.87	0.105
AST (IU/L)	44.87±35.52	26.88±13.63	0.005
ALB (g/L)	41.49±4.55	41.08±4.70	0.631
Diameter of tumor (cm)	5.88±3.52	8.48±3.53	<0.001
≤5	158(53.4%)	5(15.6%)	0.578
>5	138(46.6%)	27(84.4%)	0.578
Number of nodules:1/2/3/>3 (n)	249:30:14:3	27:2:3:-	<0.001

Comparing the primary liver cancer patients with the benign hepatic lesion patients, the median tumor size was 5.88±3.52, range from 2.5 cm to 11.3 cm, in the primary liver cancer patients compared with a median tumor size of 8.48 ± 3.53 cm, range from 3.8 cm to 13.7 cm, in the benign hepatic lesion patients. Higher platelet levels (10^9^/L) were noted in the benign hepatic lesion patients than in the primary liver cancer patients (188.91±67.10 *vs*. 145.40±71.96; *p*=0.001). Aspartate aminotransferase (AST) levels (IU/L) were higher in the primary liver cancer patients than in the benign lesion patients (44.87±35.52 *vs*. 26.88±13.63; *p*=0.005). The mean size of malignant tumors was 5.88±3.52 cm, while the mean size of benign lesions was 8.48±3.53, *p* < 0.001. In addition, the rate of nodule involvement was significantly different between the malignant patients and benign patients at 249:30:14:3 *vs*. 27:2:3:-; *p* < 0.001. (Table 1)

The baseline data for the patients who were diagnosed with HCC or ICC and their tumor characteristics are presented in Table 2. The median tumor size was 5.70±3.61 cm, range from 1.8 cm to 9.4 cm, for HCC and 6.51±3.08 cm, range from 3.1 cm to 10.3 cm, for ICC. Patients who were pathologically confirmed with HCC or ICC showed no significant differences in etiology, Child-Pugh class, white blood cell count, total bilirubin level, albumin level, and the number of tumors. The hemoglobin level was higher in the HCC patients (143.96±19.49 *vs*. 133.05±17.18, *p* < 0.001). The platelet level was lower in the HCC patients (140.89 ± 72.69 *vs*. 161.75±67.26, *p* = 0.040). The alanine aminotransferase (ALT) and AST levels were both higher in the HCC patients than in the ICC patients (47.74±43.49 *vs*. 35.00±29.87, *p* = 0.028; 47.58±37.58 *vs*. 35.05±24.60, *p* = 0.012, respectively). The level of alpha-fetoprotein (AFP) was higher in the HCC group (481.25±531.70 *vs*. 66.27±215.02; *p* = 0.002), while the CA19-9 level was higher in the ICC group (336.70±411.81 *vs*. 50.44±179.65; *p* = 0.012). The average tumor size did not differ significantly between the HCC and ICC patients; however, when we divided those patients into ≤5 cm and > 5 cm subgroups, we noticed that the ICC patients had a higher ratio of tumors >5 cm: 39/64 (60.9%) *vs*. 99/232 (42.7%), *p* = 0.011. In addition, more HCC patients had Ishak scores ≥5: 153/232 (65.9%) *vs*. 14/64 (21.9%), *p* < 0.001.

**Table 2:** The baseline of patients who were diagnosed HCC or ICC and tumor characteristics

Variables	HCC(n=232)	ICC(n=64)	*p*
Age(years)	51.04±11.49	59.38±9.42	< 0.001
Male/female(n)	202:30	43:21	0.001
Etiology (n):			
HBV	225(97.0%)	44(68.8%)	
HCV	0(0.0%)	1(1.6%)	
Hepatolithiasis	0(0.0%)	5(7.8%)	
Ethanol	0(0.0%)	7(10.9%)	
Parasitization	0(0.0%)	1(1.6%)	
Cryptogenetic	7(3.0%)	6(9.4%)	
Child-Pugh A	232(100%)	64(100%)	1.000
Hemoglobin(g/l)	143.96±19.49	133.05±17.18	< 0.001
Platelets (10^9^/L)	140.89±72.69	161.75±67.26	0.040
White blood cell (10^9^/L)	6.23±5.81	6.52±2.20	0.688
Total bilirubin (umol/L)	15.78±12.14	15.84±11.50	0.971
ALT (IU/L)	47.74±43.49	35.00±29.87	0.028
AST (IU/L)	47.58±37.58	35.05±24.60	0.012
ALB (g/L)	41.64±4.53	40.97±4.66	0.307
AFP (ng/ml)	481.25±531.70	66.27±215.02	0.002
CA19-9 (U/ml)	50.44±179.65	336.70±411.81	0.012
Diameter of tumor (cm)	5.70±3.61	6.51±3.08	0.102
≤ 5	133(57.3%)	25(39.1%)	0.011
> 5	99(42.7%)	39(60.9%)	0.011
Number of nodules:1/2/3/>3 (n)	194:24:12:2	55:6:2:1	0.858
Ishark score			
< 5	79(34.1%)	50(78.1%)	< 0.001
≥ 5	153(65.9%)	14(21.9%)	< 0.001

### US characteristics of primary liver cancer and benign hepatic lesion

**Figure 1:**
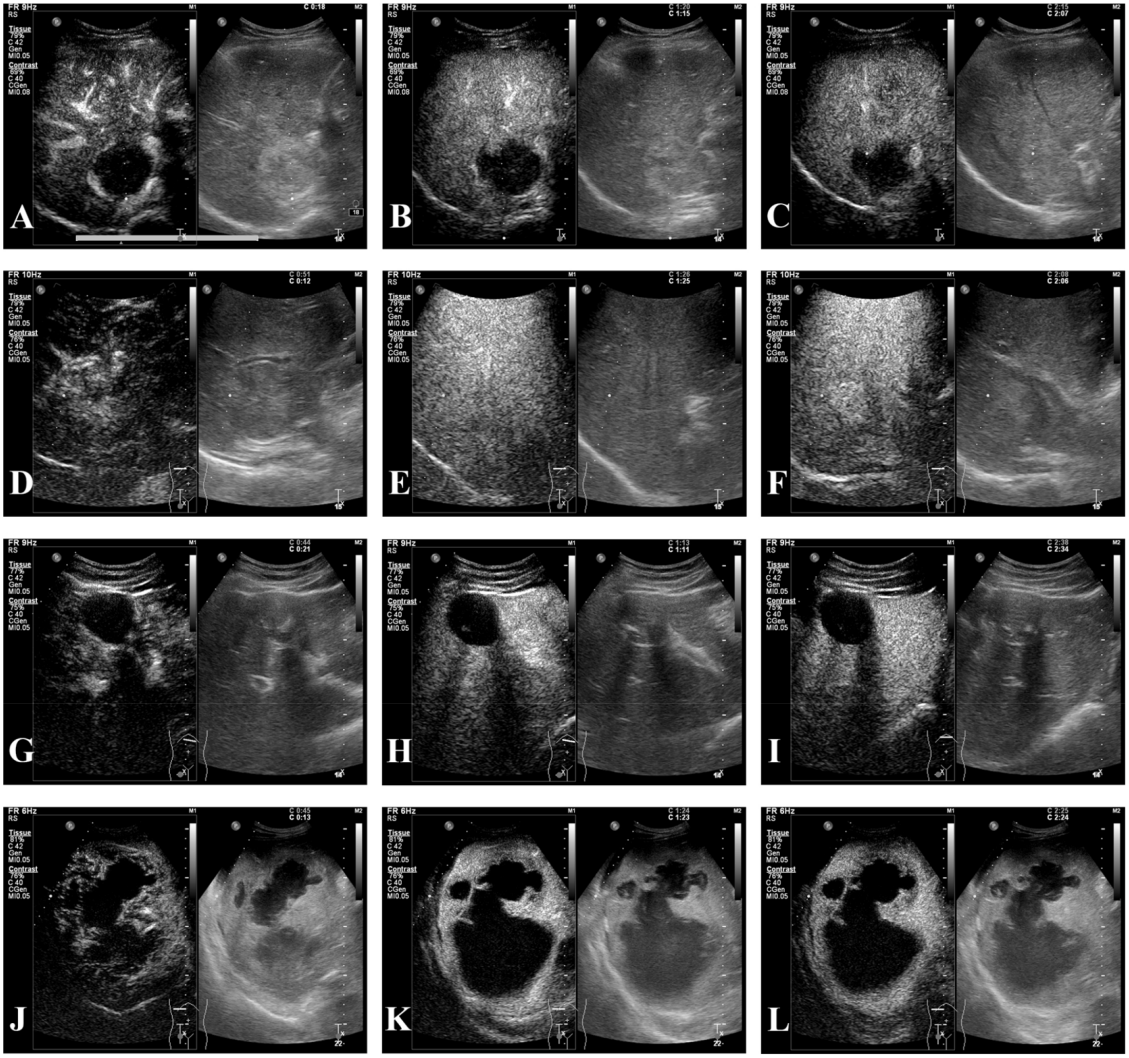
CEUS of hepatic benign lesions **(A-C)** A 46-year-old female patient with pathologically proven hemangioma which was 4.8cm in the right lobe and thrombosis inside the lesion; (A) peripheral hyperenhancement in arterial phase; (B-C) hyperenhancement in portal and delayed phases, and irregular patchy nonenhancement area inside the tumor was seen in all phases; **(D-F)** a 29-year-old male patient with pathologically confirmed focal nodular hyperplasia (FNH) which was 5.0cm in the right lobe; (D) hyperenhancement from the core to periphery in arterial phase; (E-F) peripheral hyperenhancement in portal and delayed phases, and central area presenting hypoenhancement; **(G-I)** a 35-year-old male patient with pathologically confirmed hepatic echinococcosis which was 4.4cm in the left lobe and nonenhancement in central area. **(J-L)** a 51-year-old male patient with pathogen test of drainage; (J) peripheral hyperenhancement in arterial phase; (K-L) isoenhancement to hypoenhancement in portal and delayed phases.

In conventional US (in Table 3), the constituent ratio of the baseline pattern differed between primary hepatic cancer and benign lesions (*p* < 0.001). Specifically, more primary liver cancers than benign lesions were hypoechoic (240/296, 81.1% *vs*. 11/32, 34.4%, *p* < 0.001), while benign lesions were likely to be hyperechoic (14/32, 43.8% vs.12/296, 4.1%, *p* < 0.001). Hepatic malignant tumors and benign lesions displayed different enhancement patterns in every phase: the arterial phase (*p* = 0.009), the portal phase (*p* < 0.001) and the delayed phase (*p* < 0.001). In the arterial phase, the primary differences related to the homogeneous and partial hyperenhancement of malignant tumors and benign lesions, 188/296, 63.5% *vs*. 13/32, 40.6% and *p* = 0.012; 64/296, 21.6% *vs*. 15/32, 46.9% and *p* = 0.002, respectively. In the portal and delayed phases, benign nodules still showed hyperenhancement 15/32, 46.9% *vs*. 0/296, 0.0% (*p* < 0.001); in the delayed phase, the rates were 14/32, 43.7% *vs*. 0/296, 0.0% (*p* < 0.001), but the benign nodules showed a much lower rate of isoenhancement and hypoenhancement than the malignant tumors (in the portal phase: 17/32, 53.2% *vs*. 296/296, 100% and *p* < 0.001; in the delayed phase: 18/32, 56.3% *vs*. 296/296 100% and *p* < 0.001). CEUS images for benign hepatic lesions were showed in Figure 1.

**Table 3:** Utrasonographic appearance of Primary liver cancer and benign lesions at baseline and contrast enhance ultrasound

	Primary liver cancer(n=296)	Benign lesions(n=32)	*P*
Baseline pattern			< 0.001
Hyperechoic, n	12(4.1%)	14(43.8%)	< 0.001
Isoechoic, n	5(1.7%)	1(3.1%)	0.463
Hypoechoic, n	240(81.1%)	11(34.4%)	< 0.001
Heterogeneous, n	39(13.2%)	6(18.8%)	0.415
Arterial phase			0.009
Homogeneously hyperenhanced, n	188(63.5%)	13(40.6%)	0.012
Partially hyperenhanced, n	64(21.6%)	15(46.9%)	0.002
Peripherally hyperenhanced, n	44(14.9%)	4(12.5%)	1.000
Isoenhanced, n	0	0	
Hypoenhanced, n	0	0	
Portal phase			< 0.001
Homogeneously hyperenhanced, n	0	1(3.1%)	0.098
Partially hyperenhanced, n	0	14(43.8%)	< 0.001
Peripherally hyperenhanced, n	0	0	
Isoenhanced, n	117(39.5%)	10(31.3%)	0.361
Hypoenhanced, n	179(60.5%)	7(21.9%)	< 0.001
Delayed phase			< 0.001
Homogeneously hyperenhanced, n	0	1(3.1%)	0.098
Partially hyperenhanced, n	0	13(40.6%)	< 0.001
Peripherally hyperenhanced, n	0	0	
Isoenhanced, n	2(0.7%)	11(34.4%)	< 0.001
Hypoenhanced, n	294(99.3%)	7(21.9%)	< 0.001

### Influence of size on US and CEUS diagnosis of HCC and ICC

In conventional US, the imaging patterns differed between the tumor size ≤5 cm and > 5 cm HCC subgroups (*p* < 0.001). Six out of 133 (4.51%) HCC patients with a tumor size ≤5 cm showed a heterogeneous pattern, which was lower than the rate for the HCC patients with tumor size >5 cm (6/133, 4.51% *vs*. 22/99, 22.2%, *p* < 0.001). The difference in this rate was not significant between the subgroups of ICC patients with tumor size ≤5 cm and >5 cm or between the HCC and ICC patients (Table 4).

**Table 4:** The influence of tumor size on utrasonographic appearance of HCC and ICC at baseline and contrast enhance ultrasound

	HCC				ICC				P^*^
	**Total(n=232)**	**≤5(n=133)**	**>5(n=99)**	P^**^	**Total(n=64)**	**≤5(n=25)**	**>5(n=39)**	P^***^	
Baseline pattern				<0.001				0.761	0.101
Hyperechoic, n	12(5.2%)	8(6.0%)	4(4.0%)		0	0	0		
Isoechoic, n	3(1.3%)	3(2.3%)	0(0.0%)		2(3.1%)	0	2(5.1%)		
Hypoechoic, n	189(81.5%)	116(87.2%)	73(73.7%)		51(79.7%)	21(84.0%)	30(76.9%)		
Heterogeneous, n	28(12.1%)	6(4.5%)	22(22.2%)	<0.001	11(17.2%)	4(16.0%)	7(17.9%)	1.000	
Arterial phase				<0.001				<0.001	<0.001
Homogeneously hyperenhanced, n	168(72.4%)	116(87.2%)	52(52.5%)	<0.001	20(31.3%)	16(64.0%)	4(10.3%)	<0.001	<0.001
Partially hyperenhanced, n	53(22.8%)	10(7.5%)	43(43.4%)	<0.001	11(17.2%)	2(8.0%)	9(23.1%)	<0.001	0.330
Peripherally hyperenhanced, n	11(4.7%)	7(5.3%)	4(4.0%)	0.762	33(51.6%)	7(28.0%)	26(66.7%)	0.004	<0.001
Isoenhanced, n	0	0	0		0	0	0		
Hypoenhanced, n	0	0	0		0	0	0		
Portal phase				0.690				0.417	<0.001
Homogeneously hyperenhanced, n	0	0	0		0	0	0		
Partially hyperenhanced, n	0	0	0		0	0	0		
Peripherally hyperenhanced, n	0	0	0		0	0	0		
Isoenhanced, n	110(47.4%)	65(48.9%)	45(45.5%)		7(10.9%)	4(16.0%)	3(7.7%)		
Hypoenhanced, n	122(52.6%)	68(51.1%)	54(54.5%)		57(89.1%)	21(84.0%)	36(92.3%)		
Delayed phase				0.509					1.000
Homogeneously hyperenhanced, n	0	0	0		0	0	0		
Partially hyperenhanced, n	0	0	0		0	0	0		
Peripherally hyperenhanced, n	0	0	0		0	0	0		
Isoenhanced, n	2(0.9%)	2(1.5%)	0		0	0	0		
Hypoenhanced, n	230(99.1%)	131(98.5%)	99(100%)		64(100%)	25(100%)	39(100%)		

^*^Statistical results of comparing HCC and ICC groups ; ^**^ Statistical results of comparing subgroups of different tumor size in HCC group; ^***^ Statistical results of comparing subgroups of different tumor size in ICC group.

In CEUS, the enhancement patterns in the arterial phase were different for HCC and ICC (*p* < 0.001). Of the HCC tumors, 168/232 (72.4%) displayed homogeneous hyperenhancement, while 20/64 (31.3%) of the ICC tumors showed homogeneous hyperenhancement (*p* < 0.001). Among the ICC tumors, 33/64 (51.6%) showed peripheral hyperenhancement, but only 11/232 (4.7%) HCCs displayed peripheral enhancement. In HCC tumor size subgroups, when the tumors were smaller than or equal to 5 cm, homogeneous hyperenhancement was more frequent (116/133, 87.2% *vs*. 52/99, 52.5%, *p* < 0.001), whereas partial hyperenhancement was more common in HCCs larger than 5 cm (43/99, 43.4% *vs*. 10/133, 7.5%, *p* < 0.001). ICCs less than or equal to 5 cm displayed more common homogeneous hyperenhancement (16/25, 64.0% *vs*. 4/39, 10.3%), while peripheral hyperenhancement was more common in the subgroup of ICCs larger than 5 cm (26/39, 66.7% *vs*. 7/25, 28.0%, *p* < 0.001). In the portal phase, although differences between the whole HCC and ICC groups were observed (*p* < 0.001), in terms of nodule size effect, the statistical analysis did not reveal significant results within the subgroups. In the delayed phase, almost all the lesions (HCC and ICC, 99.1% and 100%) presented with hypoenhancement.

### Influence of cirrhosis on the use of US to diagnose HCC and ICC

With the exception of the differences described above, the statistical analysis did not reveal significant additional differences between HCC and ICC against different hepatic backgrounds using conventional US and CEUS. However, our results showed that although no statistic difference was found, there was a higher proportion of homogeneous hyperenhancement in ICC patients with Ishak scores ≥ 5, 6/14 (42.9%) *vs*.14/50 (28.0%), and peripheral hyperenhancement was more frequently observed in ICC patients with Ishak scores < 5 during the arterial phase. In the portal and delayed phases, no significant results were found (in Table 5).

**Table 5:** The influence of cirrhosis on utrasonographic appearance of HCC and ICC at baseline and contrast enhance ultrasound

	HCC				ICC				^*^P
	**Total(n=232)**	**Ishak< 5(n=79)**	**Ishak ≥ 5(n=153)**	^**^P	**Total(n=64)**	**Ishak<5(n=50)**	**Ishak ≥ 5(n=14)**	^***^P	
Baseline pattern				0.192				0.434	0.101
Hyperechoic, n	12(5.2%)	4(5.1%)	8(5.2%)		0	0	0		
Isoechoic, n	3(1.3%)	0	3(2.0%)		2(3.1%)	1(2.0%)	1(7.1%)		
Hypoechoic, n	189(81.5%)	61(77.2%)	128(83.7%)		51(79.7%)	41(82.0%)	10(71.4%)		
Heterogeneous, n	28(12.1%)	14(17.7%)	14(9.2%)		11(17.2%)	8(16%)	3(21.4%)		
Arterial phase				0.264					0.360
Homogeneously hyperenhanced, n	168(72.4%)	52(65.8%)	116(75.8%)	0.107	20(31.3%)	14(28.0%)	6(42.9%)	0.336	
Partially hyperenhanced, n	53(22.8%)	22(27.8%)	31(20.3%)	0.192	11(17.2%)	8(16.0%)	3(21.4%)	0.693	
Peripherally hyperenhanced, n	11(4.7%)	5(6.3%)	6(3.9%)	0.516	33(51.6%)	28(56.0%)	5(35.7%)	0.179	
Isoenhanced, n	0	0	0		0	0	0		
Hypoenhanced, n	0	0	0		0	0	0		
Portal phase				0.495				1.000	<0.001
Homogeneously hyperenhanced, n	0	0	0		0	0	0		
Partially hyperenhanced, n	0	0	0		0	0	0		
Peripherally hyperenhanced, n	0	0	0		0	0	0		
Isoenhanced, n	110(47.4%)	35(44.3%)	75(49.0%)		7(10.9%)	6(12.0%)	1(7.1%)		
Hypoenhanced, n	122(52.6%)	44(55.7%)	78(51.0%)		57(89.1%)	44(88.0%)	13(92.9%)		
Delayed phase				0.549					1.000
Homogeneously hyperenhanced, n	0	0	0		0	0	0		
Partially hyperenhanced, n	0	0	0		0	0	0		
Peripherally hyperenhanced, n	0	0	0		0	0	0		
Isoenhanced, n	2(0.9%)	0	2(1.3%)		0	0	0		
Hypoenhanced, n	230(99.1%)	79(100%)	151(98.7%)		64(100%)	50(100%)	14(100%)		

^*^Statistical results of comparing HCC and ICC groups; ^**^ Statistical results of comparing subgroups of different hepatic background in HCC group; ^***^ Statistical results of comparing subgroups of different hepatic background in ICC group.

**Figure 2:**
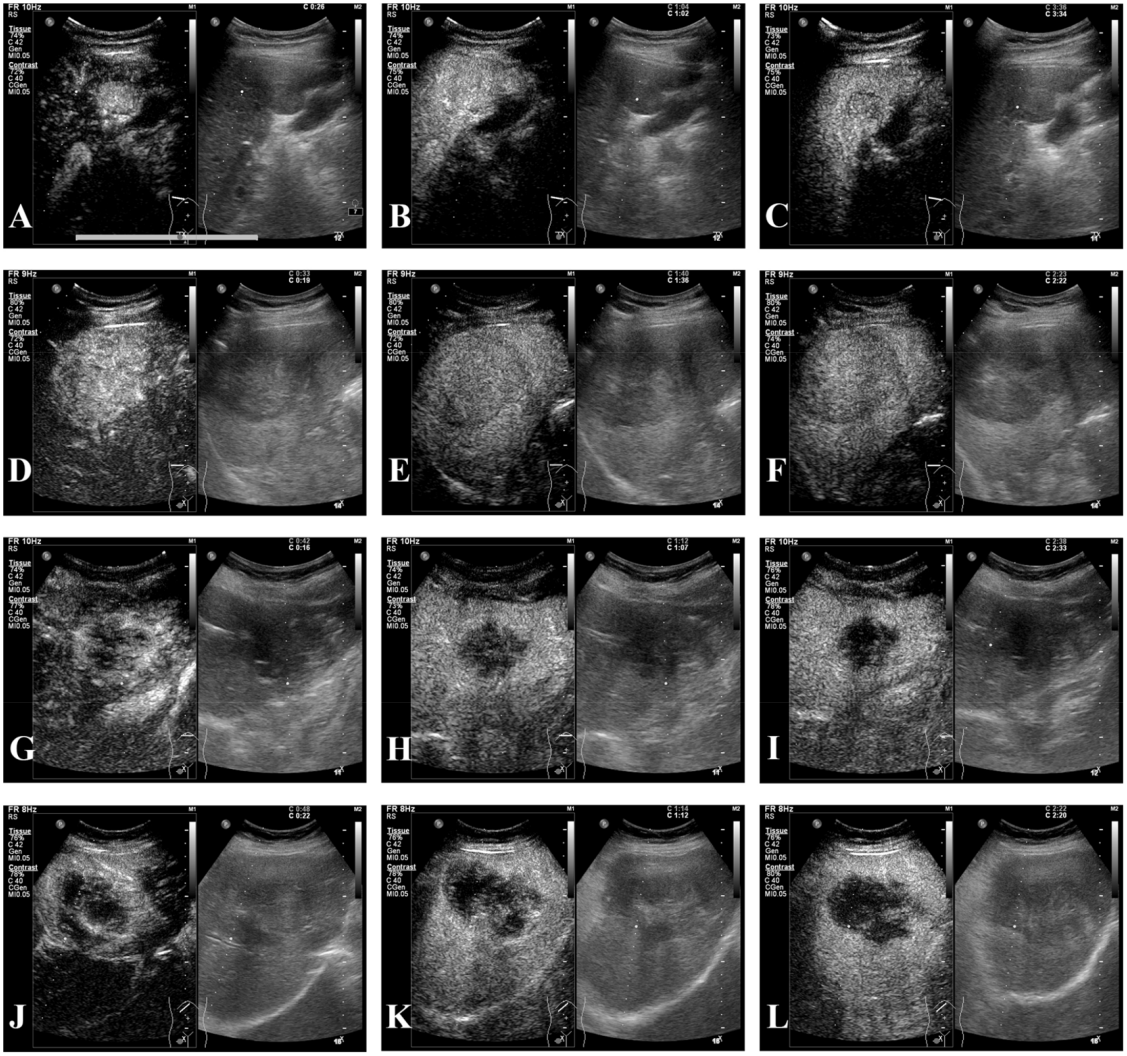
CEUS of HCC and ICC **(A-C)** A 59-year-old male patient with pathologically diagnosed HCC and cirrhosis whose tumor was 2.7cm; (A) homogeneous hyperenhancement in arterial phase; (B) isoenhancement in portal phase; (C) hypoenhancement in delayed phase; **(D-F)** a 56-year-old male patient with pathologically proven HCC and cirrhotic and the tumor was 7.9cm; (D) homogeneous hyperenhancement in arterial phase; (E-F) hypoenhancement in portal and delayed phases; **(G-I)** this was a female patient of 46 years old, who was pathologically diagnosed with ICC of 4.7cm; (G) peripheral hyperenhancement in arterial phase; (H-I) hypoenhancement in portal and delayed phases; **(J-L)** a 53-year-old female patient with pathologically confirmed ICC of 8.6cm; (J) partial hyperenhancement in arterial phase; (K-L) hypoenhancement in portal and delayed phases.

A comparison of the CEUS enhancement patters for HCC and ICC to nodule size and cirrhotic background is presented in Table 6. In the arterial phase, when comparing the ≤5 cm and >5 cm subgroups of HCC and ICC, both HCC and ICC showed a high rate of homogeneous hyperenhancement in the ≤5 cm subgroup (116/133, 87.2% *vs*. 16/25, 64.0% and *p* = 0.008); however, in the >5 cm subgroup, the rate of homogeneous hyperenhancement was much lower in the case of ICC (52/99, 52.2% *vs.* 4/39, 10.3% and *p* < 0.001). The rate of peripheral hyperenhancement was low when the tumors were 5 cm or smaller, 7/133, 5.3% *vs*.7/25, 28.0% and *p* = 0.002. When the tumors were larger than 5 cm, the rates of peripheral hyperenhancement in HCC *vs.* ICC were 4/99, 4% *vs*. 26/39, 66.7% and *p* < 0.001. In the portal phase, the ≤5 cm subgroups of HCC and ICC displayed isoenhancement and hypoenhancement rates of 65/133, 48.9% *vs*. 4/25, 16.0% (*p* = 0.002) and 68/133, 51.1% *vs*. 21/25, 84.0% (*p* = 0.002), respectively; the >5 cm subgroup displayed rates of 45/99, 45.5% *vs*. 3/39, 7.7% (*p* < 0.001) and 54/99, 54.5% *vs*. 36/39, 92.3% (*p* < 0.001), respectively. In terms of cirrhotic background, in the arterial phase, the rate of HCC *vs*. ICC tumors in the Ishak score <5 subgroups with homogeneous hyperenhancement and peripheral hyperenhancement was 52/79, 65.8% *vs*. 14/50, 28.0% (*p* < 0.001) and 5/79, 6.3% vs. 28/50, 56.0% (*p* < 0.001), respectively; for the Ishak score ≥5 subgroups, the rates were 116/153, 75.8% vs. 6/14, 42.9% (*p* = 0.023), and 6/153, 3.9% *vs*. 5/14, 35.7% (*p* = 0.001). In the portal phase, the differences between the Ishak <5 and ≥5 subgroups of HCC and ICC that showed isoenhancement were 35/79, 44.3% *vs*. 6/50, 12.0% (*p* < 0.001) and 75/153, 49.0% *vs*. 1/14, 7.1% (*p* = 0.003), respectively, while 44/79, 55.7% vs. 44/50, 88% (*p*<0.001) and 78/153, 51.0% *vs*. 13/14, 92.9% (*p* = 0.003), respectively, showed hypoenhancement. CEUS images for HCC and ICC were presented in Figure 2.

**Table 6:** The comparison of enhancement patterns between HCC and ICC

	HCC *vs*. ICC	
**≤5cm**		
**Arterial phase**	116:10:7:0:0 *vs*. 16:2:7:0:0	*p* = 0.003
Homogeneously hyperenhanced	116/133, 87.2% *vs*.16/25, 64.0%	*p* = 0.008
Partially hyperenhanced	10/133, 7.5% *vs*.2/25, 8.0%	*p* = 1.000
Peripherally hyperenhanced	7/133, 5.3% *vs*.7/25, 28.0%	*p* = 0.002
**Portal phase**	0:0:0:65:68 *vs*. 0:0:0:4:21	*p* = 0.004
Isoenhanced	65/133, 48.9% *vs*. 68/133, 51.5%	*p* = 0.002
Hypoenhanced	68/133, 51.1% *vs*. 65/133, 48.9%	*p* = 0.002
**>5cm**		
**Arterial phase**	52:43:4:0:0 *vs*. 4:9:26:0:0	*p* < 0.001
Homogeneously hyperenhanced	52/99, 52.2% *vs.* 4/39, 10.3%	*p* < 0.001
Partially hyperenhanced	43/99, 43.4% *vs*. 9/39, 23.1%	*p* = 0.026
Peripherally hyperenhanced	4/99, 4.0% *vs*. 26/39, 66.7%	*p* < 0.001
**Portal phase**	0:0:0:45:54 *vs*. 0:0:0:3:36	*p* < 0.001
Isoenhanced	45/99, 45.5% *vs*. 3/39, 7.7%	*p* < 0.001
Hypoenhanced	54/99, 54.5% *vs*. 36/39, 92.3%	*p* < 0.001
**Ishak <5**		
**Arterial phase**	52:22:5:0:0 *vs*. 14:8:28:0:0	*p* < 0.001
Homogeneously hyperenhanced	52/79, 65.8% *vs*. 14/50, 28.0%	*p* < 0.001
Partially hyperenhanced	22/79, 27.8% *vs*. 8/50, 16%	*p* = 0.121
Peripherally hyperenhanced	5/79, 6.3% vs. 28/50, 56.0%	*p* < 0.001
**Portal phase**	0:0:0:35:44 *vs*. 0:0:0:6:44	*p* < 0.001
Isoenhanced	35/79, 44.3% *vs*. 6/50, 12.0%	*p* < 0.001
Hypoenhanced	44/79, 55.7% *vs*. 44/50, 88%	*p* < 0.001
**Ishak ≥5**		
**Arterial phase**	116:31:6:0:0 *vs*. 6:3:5:0:0	*p* = 0.001
Homogeneously hyperenhanced	116/153, 75.8% vs. 6/14, 42.9%	*p* = 0.023
Partially hyperenhanced	31/153, 20.3% *vs*. 3/14, 21.4	*p* = 1.000
Peripherally hyperenhanced	6/153, 3.9% *vs*. 5/14, 35.7%	*p* = 0.001
**Portal phase**	0:0:0:75:78 *vs*. 0:0:0:7:13	*p* = 0.003
Isoenhanced	75/153, 49.0% *vs*. 1/14, 7.1%	*p* = 0.003
Hypoenhanced	78/153, 51.0% *vs*. 13/14, 92.9%	*p* = 0.003

## DISCUSSION

CEUS were first used to identify focal liver lesions 10 years ago and are currently used in daily clinical practice in many countries. The ultrasound contrast agent sulfur-hexafluoride filled microbubbles (SonoVue, Bracco, Milan, Italy), contributes to its wide application for diagnosing hepatic lesions because SonoVue only distributes intravascularly, without any passage into the interstitial space, which allows the analysis of the entire liver parenchyma [2]. In comparison, the contrast agent used for CT and MRI can leak into the interstitium of lesions, resulting in long-lasting enhancement [15]. Thus, CEUS can display clearer intrahepatic vascular characteristics and more information during delayed phase.

Limited studies have analyzed the different CEUS characteristics of primary liver cancer and benign hepatic lesions. Imaging findings similar to those of malignant tumors may be observed in some special types of benign nodules, such as hemangiomas with high flow (or shunt) or thrombosed hemangiomas; FNH with centrally located scars, which can present hypoenhancement in the portal and delayed phases; abscess during tumor development, etc. [4, 16]. In our study, although CEUS found significant differences between malignant and benign hepatic lesions in the arterial phase, its practical function may be limited. However, in the portal and delayed phases, when dynamic changes in the lesion appear on imaging, obvious differences were noticed. This result is consistent with that of a previous study [17]. W-T Kong et al. selected focal liver lesions that all presented quick enhancement in the arterial phase and quick wash-out in the portal phase on CEUS. They found that the CEUS features in the arterial phase may not provide enough information to differentiate between malignant and benign liver masses, but it could differentiate malignant and benign characteristics in the portal and delayed phases [18]. Therefore, we conclude that CEUS could be helpful in the differential diagnosis of hepatic malignant and benign lesions and that all phases and dynamic images of a tumor need to be analyzed.

Regarding distinguishing between HCC and ICC, in 2010, the study of Vilana et al.[8] found that ICC with a cirrhotic hepatic background and HCC had similar CEUS enhancement characteristics: CEUS imaging presented homogeneous hyperenhancement in the arterial phase followed by hypoenhancement in the portal and delayed phases. Therefore, CEUS was dropped from the list of diagnostic methods for nodules with a cirrhotic background in the updated guidelines of the AASLD in 2011 [9] and the guidelines of the EASL in 2012 [10]. However, this removal has led to numerous controversial arguments [11, 14, 19–21]. The various nature of ICC enhancement patterns of ICC has been widely discussed [8, 11, 13, 14, 20, 21]. To the best of our knowledge, our study is one of the largest series to analyze ICCs with both different sizes and hepatic backgrounds. In this study, during the arterial phase, we noticed that high rate of ICCs ≤5 cm displayed homogeneous enhancement, which is in agreement with previous studies [8, 11, 13, 14, 21]. When ICC tumors are small, they have abundant tumor tissue and minimal fibrous tissue; thus, their enhancement pattern can be homogeneous [14]. We found that 33/64 (51.6%) ICCs showed peripheral hyperenhancement, especially those that were larger than 5 cm (26/39, 66.7%). This could contribute to larger size in our study (median size 6.51±3.08 cm; range from 3.1 cm to 10.3 cm) and the higher proportion of nodules more than 5 cm (39/64, 60.9%). During the portal phase, a hypoenhancement pattern was more frequently observed in ICC, particular when tumors were >5 cm (36/39, 92.3%); this conclusion is similar to those of previous studies [14, 20]. When hepatic background was considered, in the arterial phase, both ICC and HCC demonstrated homogeneous hyperenhancement against a cirrhotic background. This could contribute to the developing number of arterial branches and small vessels [12]. In the arterial phase, the number of ICCs that displayed homogeneous hyperenhancement against a cirrhotic background was 6/14 (42.9%). However, Galassi, M., et al. reported a 68% (17/25) rate of homogeneous hyperenhancement in the arterial phase in ICC [21], and Vilana et al. reported a 47.6% rate of homogeneous hyperenhancement in ICC. This difference may be explained by tumor size. The median diameter of the tumors in our study was 6.51±3.08 cm, while in the studies by Galassi, M., et al. and Vilana et al., the median diameters were 3.04 ± 1.16 cm and 3.2 cm, respectively. As far as we are concerned, in the arterial phase, although HCC showed more common homogeneous hyperenhancement and peripheral hyperenhancement was more likely in ICC, these statistical differences may not have much clinical significance for distinguishing HCC from ICC in tumors ≤5 cm. In the portal phase, when ICCs were larger than 5 cm, a more dramatic wash-out was observed: 7.7% displayed isoenhancement and 92.3 showed hypoenhancement, compared with 51.5% and 48.9%, respectively, when the tumor was ≤5 cm. However, against different hepatic backgrounds, this difference was not obvious. Regardless of the hepatic background, ICC displayed a dramatic wash-out. Hence, peripheral hyperenhancement in ICC may still have a differential diagnostic value, and imaging features in the portal and delayed phases also could have a differential diagnostic value. However, when the ICC was less than 5 cm and against a cirrhotic background, it could be difficult to distinguish from HCC using CEUS alone; thus, other imaging examinations and clinical information should be comprehensively considered.

The purpose of distinguishing primary liver cancer and hepatic benign lesions is to perform suitable treatment. Patients with benign lesions may avoid surgical procedures or may only need internal care. Additionally, most studies set 3 cm as a cutoff for differentiating HCC from ICC, since their very similar features of CEUS imaging [8, 11, 14, 20]. In this study, we considered 5 cm as a cutoff point because both literature reports and clinical observation have shown poor surveillance of high-risk patients. In studies of HCC surveillance in the United States, less than 20% of cirrhotic patients developed HCC received regular surveillance [22]. Hence, tumors may be relatively large when they are first found. More importantly, before surgical treatment, it is vital for surgeons to differentially diagnose malignant tumors. If it is technically feasible and there is no vascular invasion, a patient with solitary HCC or ICC, even with tumors larger than 5 cm, could benefit from hepatic resection [23, 24]. However, when a patient is diagnosed with a malignant hepatic tumor, it is very prudent to differentiate between HCC and ICC, especially for patients whose tumors are smaller than or equal to 5 cm and those who meet the Milan criteria and are willing to wait for a liver transplantation. HCC patients who meet the Milan criteria benefit from liver transplantation [23]. In contrast, in patients with ICC their overall survival and disease free survival may not benefit from liver transplantation, and they may take the risks of intraoperative and perioperative complications, or only carefully selected patients and those with very early ICC may benefit from liver transplantation. [25–27]. Jung DH et al. and Togashi J et al. found incidentally diagnosed ICC after liver transplantation. [27, 28]. Jung DH et al., in their recent study, reported that the prognosis of patients incidentally diagnosed with ICC after liver transplantation was as poor as that of ICC patients who were treated by hepatic resection with propensity score-matching. Although CEUS was dropped from the list of diagnostic methods for nodules with cirrhotic background in the updated guidelines of AASLD in 2011 [9] and the guidelines of EASL in 2012 [10], CEUS offers the advantages of real-time observation, low cost, accessibility, and freedom from radiation risks; additionally, given the high prevalence of hepatitis B virus, which leads to hepatic cirrhosis in China [29], CEUS may provide extra information about lesions and an efficient technique for surveillance and diagnosis. CEUS cannot replace CT or MRI for the diagnosis of hepatic masses, or vice versa. Because each of these techniques has its own unique advantages, when one fails to identify a lesion, adding another imaging examination could be useful for examining other aspects. Therefore, CEUS could be helpful in preoperative determination.

Several studies have analyzed and presented detailed CEUS information for differentially diagnosing ICC. In our study, we presented CEUS imaging information regarding homogeneous, partial, peripheral, iso- and hypo- enhancement. These simplified patterns may be easier for clinicians to identify.

There are still some limitations of this study. First, our results need to be validated in prospective studies, which is a common drawback of retrospective research. Second, the proportions of patients with ICC and benign tumors were relatively small given the inclusion criteria of this study, and the fact that the incidence of ICC is much lower than that of HCC. For hepatic benign tumors, although the incidence of benign tumors was not as low as that of hepatic malignant cancers, the numbers of patients who met the surgical indications was still small. Hence the potential bias should be decreased by including larger patients in future studies. Third, when differentially diagnosing malignant and benign lesions, hepatic cirrhosis and size may also need to be taken into consideration in future studies.

In conclusion, the size of the tumor and the hepatic background should be taken into consideration when distinguishing HCC and ICC before surgery; however, CEUS is a helpful tool for differentiating hepatic malignant and benign lesions. For patients who require surgical treatment, CEUS may help for surgical decision making.

## MATERIALS AND METHODS

### Patients

We retrospectively reviewed patients who were diagnosed with hepatic masses and who had undergone CEUS at the department of liver surgery and liver transplantation center, West China Hospital of Sichuan University between January 2010 and December 2015. The study protocol and patient consent procedure were approved by the Biomedical Research Ethics Committee of West China Hospital of Sichuan University. All patients signed their written informed consent in accordance with the ethical guidelines of the Declaration of Helsinki before participating in this study.

The following inclusion criteria were applied:

Exclusion criteria were as follows:

Since CEUS cannot scan multiple nodules simultaneously after one injection of contrast agent, when the nodules were not in the same scan plane, a per-patient analysis was performed; that is, when a patient had more than one solid lesion in the liver, the largest one was measured and investigated.

### Image acquisition

US examinations were performed by four experienced sonographers with approximately 8, 10, 12, and 32 years of experience in liver ultrasound examination. We performed CEUS examinations by an iU22 ultrasound system (Philips Royal Electronic Corporation, The Netherlands) with a C5-1 transducer. Real-time contrast imaging setting was used with a low mechanic index of 0.2 to avoid the microbubbles disruption. In CEUS study, a volume of 2.4-4.8 mL of contrast agent SonoVue (Bracco of Italy) was quickly injected through the antecubital vein. The pipe was washed with 5 mL of 0.9% sodium chloride solution. A timer began as soon as the contrast agent was injected, and continuous observations occurred for at least five minutes. The whole phases of contrast enhancement was recorded and analyzed, which consisted of arterial phase (0-30s after injection), the portal phase (31-120s) and delay phase (>120s after injection), according to the European Federation of Societies for Ultrasound in Medicine and Biology (EFSUMB) recommendation [16].

### Image analysis

Four sonographers with approximately 8, 10, 12 and 30 years of experience in abdominal US performed the ultrasonography. The sonographers were aware that the patients were at risk of developing HCC but did not have access to additional information, *e.g.*, AFP and CA19-9 levels. In cases of disagreement, the sonographers engaged in joint discussions until a consensus was reached.

Conventional US examination was performed for all enrolled patients before CEUS and used to describe the size, location and number of lesions. The conventional US imaging patterns were classified as hyperechoic, isoechoic, hypoechoic and heterogeneous.

The vascular contrast patterns on CEUS were defined by comparing the enhancement behavior of the tumor with the surrounding liver parenchyma and were classified as follows:

### Statistical analysis

The baseline characteristics of the patients are expressed as the median ± SD or count and proportion. Continuous variables are expressed as the median ± SD and were compared between groups with a t test or Mann-Whitney U test for variables with an abnormal distribution. The categorical data were compared using the chi-squared test/Fisher's exact test. A conventional P value of < 0.05 was considered statistically significant. Calculations were performed with the SPSS 19.0 package (SPSS, Inc., Chicago, IL).

## Abbreviations

AASLD: American Association for the Study of Liver Diseases
AFP: alpha-fetoprotein
ALT: alanine aminotransferase
AST: aspartate aminotransferase
CEUS: contrast-enhanced ultrasound
CT: computed tomography
EASL: European Association for the Study of the Liver
EFSUMB: European Federation of Societies for Ultrasound in Medicine and Biology
FNH: focal nodular hyperplasia
HCC: hepatocellular carcinoma
ICC: intrahepatic cholangiocarcinoma
MRI: magnetic resonance imaging
US: ultrasound
